# Antidrug antibodies to adalimumab do not associate with immunologically related adverse events

**DOI:** 10.3389/fimmu.2024.1457993

**Published:** 2025-02-27

**Authors:** Sophie Tourdot, Maria-Dolores Vazquez-Abad, Donna S. Cox, Chun-Hua Cai, Karen Wang, Wuyan Zhang, Christopher Lepsy

**Affiliations:** ^1^ Pharmacokinetics, Dynamics and Metabolism, Pfizer Inc., Andover, MA, United States; ^2^ Product Research & Development, Inflammation & Immunology, Pfizer Inc., Cambridge, MA, United States; ^3^ Clinical Pharmacology, Pfizer Inc., Collegeville, PA, United States; ^4^ Clinical Pharmacology, Pfizer Inc., Groton, CT, United States; ^5^ Oncology Research & Development, Pfizer Inc., La Jolla, CA, United States; ^6^ Research & Development, Pfizer Inc., Lake Forest, IL, United States

**Keywords:** adalimumab, antidrug antibodies, hypersensitivity, immunogenicity, neutralizing antibodies, pharmacokinetic

## Abstract

**Introduction:**

Unwanted immune responses (UIRs) to biologics can negatively impact treatment efficacy and pharmacokinetics and/or induce adverse events (AEs). We characterized the UIR profile of adalimumab (ADL) using data from a phase 3, randomized, interchangeability study of reference ADL (ADL-REF; Humira^®^) and ADL biosimilar PF-06410293 in patients with rheumatoid arthritis (RA).

**Methods:**

Eligible patients (18−70 years, moderate-to-severe active RA) received ADL-REF from weeks 0−10 (lead-in period) then were randomized 1:1 to: 3 switches between PF-06410293 and ADL-REF or continuous ADL-REF treatment until week 32. As interchangeability of PF-06410293 with ADL-REF was previously demonstrated, data were combined across groups to describe the development of antidrug antibodies (ADAs) and their impact on pharmacokinetics and immunologically related AEs. Pharmacokinetic endpoints included maximum observed serum concentration (C_max_), area under serum concentration–time curve over dosing interval (AUC_tau_), time of maximum observed serum concentration (T_max_), average serum concentration (C_av_), and apparent clearance (CL/F), determined from robust pharmacokinetic sampling during weeks 30–32; and predose concentrations (C_trough_) at prespecified sampling time points. Other endpoints: patients (%) with ADA-positive and neutralizing ADA (NAb)-positive samples, time of first ADA/NAb detected, ADA titers over time, persistence of ADA/NAb, and immunologically related AEs by ADA/NAb status.

**Results:**

Of 427 randomized patients, 59% were ADA-positive, 52% had persistent ADA, 14% were NAb-positive, and 10% had persistent NAb. In most patients, ADA/NAb first developed within 16 weeks of ADL treatment regardless of pre-existing (baseline day 1) ADA. ADA/NAb titers stabilized by week 16 without boosters. C_trough_ was lower in patients with ADA-positive than ADA-negative samples throughout the study. From weeks 30–32, AUC_tau_, C_max_, and C_av_ were lower in ADA-positive than ADA-negative samples at week 30, especially in patients with ADA-positive/NAb-positive samples. Only 3% of patients had immunologically related AEs. Most were injection site and hypersensitivity reactions, and none were considered severe or serious or associated with the presence of ADA/NAb. Presence of pre-existing ADA did not increase the potential for immunologically related responses to ADL.

**Conclusions:**

Presence of ADA (with or without NAb) was associated with lower drug concentrations and faster clearance but not with the development of immunologically related AEs.

**Clinical trial registration:**

ClinicalTrials.gov, identifier NCT0423021.

## 1 Introduction

Multiple inflammatory diseases are characterized by dysregulation of tumor necrosis factor alpha (TNFα), and the development of anti-TNFα inhibitors has been a milestone in the treatment of rheumatoid arthritis (RA), inflammatory bowel disease, ankylosing spondylitis, and psoriatic arthritis. Anti-TNFα biological drugs such as adalimumab (ADL) possess known immunogenic properties which may result in the formation of antidrug antibodies (ADAs), with or without neutralizing activity (NAbs) (1, 2). The US Food and Drug Administration (FDA) have defined immunogenicity as “the propensity of a therapeutic protein product to generate an unwanted immune response to itself and to related proteins or to induce immunologically related adverse clinical events” (3). In addition, the European Medicine Agency (EMA) have defined immunogenicity as “unwanted immune reaction to a therapeutic protein” (4, 5). Hereafter, we use the term “immunogenicity” to refer to unwanted immune responses to the drug, and “immunologically related adverse events (AEs) after administration of the drug”, as defined by regulatory authorities. Hypersensitivity reactions associated with biological drugs may occur more frequently with increased use and can be classified as immediate or delayed. Immediate hypersensitivity reactions include infusion-related reactions, cytokine release reactions, type I (immunoglobulin [Ig]E and non-IgE) reactions and mixed reactions (IgE and cytokine-release). Delayed reactions include type III (serum sickness reactions) and type IV reactions (6). The formation of ADAs either during treatment with biologicals or pre-existing may contribute to an increased risk of immediate hypersensitivity reactions to some biological drugs (6).

Although it is well recognized that all therapeutic proteins have the potential to be immunogenic, the resulting consequences can vary significantly. Immunogenicity has the potential to impact pharmacokinetics (PK), efficacy, and/or safety. However, the presence of an immunogenic response may not always lead to a clinically meaningful effect. Hence, characterization of immunogenicity is key to understanding how to appropriately manage patients on these drugs.

A wide range of ADA occurrence following treatment with ADL has been reported in the literature, from 1.9% (7) to 94.5% (8, 9). This variation in percent of patients with ADA reported can be partially attributed to the methodology of ADA detection used, including sensitivity and drug- and target-tolerance of the assay (10) as well as the design and duration of study. The reporting details for the ADA assay methodology, sensitivity, tolerances, and results can be variable and not standardized across studies (11, 12). However, the development of drug-tolerant assays has enabled the measurement of ADA levels in the presence of high therapeutic drug concentrations (13), avoiding the pitfalls of potentially reporting false-negative ADA results and providing more confidence in interpretation of data. The specific impact of immunogenicity on safety can be difficult to evaluate in clinical studies that may result from inconsistent or absence of systematic safety assessments that may lead to lack of identification of immunologically related safety events. Regulatory guidelines related to immunogenicity have detailed the clinical consequences that should be evaluated in clinical trials (14, 15).

Previous studies in patients with RA suggest that those who developed ADAs against ADL during a 3-year treatment period less frequently achieved minimal disease activity or remission, and the development of ADAs with NAbs against ADL was associated with lower serum ADL concentrations or higher clearance of ADL (16, 17). Concomitant methotrexate (MTX) treatment with anti-TNFα therapies in RA has been reported to decrease the development of ADA and, hence, avoid the potential impact of decreasing circulating serum drug concentrations. It is commonly used in patients with RA undergoing anti-TNFα therapy (18).

Our recent phase 3, randomized, multi-switch clinical study demonstrated interchangeability between PF-06410293 biosimilar and reference adalimumab (ADL-REF; Humira^®^) (19). Here, we evaluated a set of data from this interchangeability study (19) to characterize the immunogenic profile of ADL (20). Importantly, in this study, ADAs were measured using validated assays with known sensitivity and drug and target tolerances, and samples were collected at multiple timepoints for measurement of ADA and PK. Furthermore, since interchangeability was established between PF-06410293 and ADL-REF, all patient data from both treatment arms were pooled for this analysis. Concurrent ADA and NAb assessment and PK sampling were performed at 9 timepoints during the study. A systematic and comprehensive safety review and evaluation was performed to identify potential immunologically related AEs, which were then assessed relative to the corresponding ADA status before the time of AE start and based on pre-existing ADA.

## 2 Materials and methods

### 2.1 Study design

The study design for this randomized, controlled clinical trial has been described previously in detail (19). Briefly, all patients received ADL-REF from weeks 0−10 (lead-in period). After the lead-in period, patients were randomized 1:1 to either 3 switches between PF-06410293 and ADL-REF or to continuous treatment with ADL-REF until the end of study at week 32. Randomization at week 10 was stratified by body weight groups (≥40–<70 kg, ≥70–<100 kg, and ≥100–≤130 kg), as body weight has previously been found to be a predictor of ADL clearance (21). All patients were dosed with 40 mg PF-06410293 or ADL-REF every 2 weeks with no dose modification (19). For this study, data from both ADL treatment arms were combined to characterize the immunogenicity of ADL.

The study was conducted in compliance with the ethical principles originating in or derived from the Declaration of Helsinki and in compliance with all International Conference on Harmonization Good Clinical Practice guidelines and local regulatory requirements. Written informed consent was obtained before any study-specific activity was performed. The final protocol, any amendments, and informed consent documentation were reviewed and approved by the institutional review board and/or independent ethics committee at each of the investigational centers participating in the study.

### 2.2 Patients

Patient eligibility criteria were described previously (19). Briefly, patients of either sex (18−70 years of age) were eligible for enrollment if they had moderately to severely active RA and were on a stable dose of MTX (19). Prior to study participation (which includes lead-in) patients did not have prior treatment with ADL.

### 2.3 Immunogenicity, pharmacokinetic, and safety endpoints

#### 2.3.1 Antidrug antibody measurements

Blood samples to determine the presence of ADA and NAb were collected predose on baseline/day 1, and at weeks 10, 16, 22, 24, 26, 28, 30, and 32, concurrently with samples for PK analysis. Additional samples for PK analyses were collected between weeks 30 and 32 and characterized by patient’s week 30 ADA and NAb status. The rationale for the timing of ADA sampling was based on the generally accepted principle that if treatment-emergent IgG ADA occurs, onset could be observed as soon as 10−14 days post dose. The half-life of human IgG antibodies (10–21 days) (22) was also taken into consideration. Thus, if IgG ADA were present at week 30, they would most likely still be circulating between weeks 30 and 32.

ADAs against ADL were detected using a validated, high‐sensitivity, drug‐ and target-tolerant electrochemiluminescent (ECL) assay with an acid dissociation step to improve drug tolerance in serum. The analytical platform was Meso Scale Discovery (MSD) and used high‐bind plates. ADA assay design was conducted in accordance with regulatory guidance (3). Human serum samples and positive and negative controls were diluted in 300 mM acetic acid to dissociate potential endogenous TNF-α interference and potential ADA and PF-06410293 complexes in samples. Acid-dissociated samples were neutralized with 1.5M Tris-base and co-incubated with biotinylated-PF-06410293 and ruthenium-labeled PF-06410293 (master mix) on a polypropylene microtiter plate. During this time, anti-ADA antibodies will bind to both the biotinylated-PF-06410293 and ruthenylated-PF-06410293 molecules to form an antibody complex bridge. After incubation, 50 μL of sample mixture was added to the wells of the Streptavidin coated MSD plate and incubated for approximately 1 hour. In the presence of tripropylamine-containing read buffer, ruthenium produces a chemiluminescent signal that is triggered when voltage is applied. The resulting chemiluminescence was measured in response unit. Data were calculated using Watson 7.4.1 Immune Response Module and presented as end point log titers (log_10_), defined as the reciprocal of the serum dilution at which the sample response would be equal to the cut point of the assay. The validation parameters of drug tolerance at the low-positive control (250 ng/mL) and high-positive control (8000 ng/mL) were 6.4 and 150 µg/mL, respectively. A minimum required dilution (by how much the sample must be diluted to avoid matrix effects) of 1:75 was used to report ADA-negative and ADA-positive cut-offs, such that samples with ADA titers of log_10_(75) or <1.88 were considered ADA-negative, and those with ADA titers ≥1.88 were considered ADA-positive. The assay sensitivity for affinity-purified anti-ADL mAb was 10.3 ng/mL (Pfizer unpublished data).

ADA-positive samples were tested for NAb using a validated cell-based assay following a tiered approach (screening and titer). Briefly, a cell line with high sensitivity to TNF-α was used. NAb to PF-06410293 will bind to the drug and restore the TNF-α induced cytotoxicity of cells. In this homogenous assay, samples, negative controls, and positive controls were pre-incubated with PF-06410293 and TNF-α before addition to cells. CellTiter Glo^TM^ was used to generate the signal by quantitating adenosine triphosphate, which is an indicator of metabolically active cells. Screening cut point factor (SCPF) was established during the full NAb validation to determine NAb-negative or NAb-positive status. NAb titer data were presented as log_10_ with the end point titer defined as the reciprocal of the serum dilution at which the sample response would be equal to the cut point of the assay. Samples falling at NAb SCPF titer were reported at minimum required dilution (log_10_(5)=0.70). Samples with NAb titers of log_10_(5) or <0.70 were considered NAb-negative, and those with NAb titers ≥0.70 were considered NAb-positive. ADA-negative samples were not tested for NAb and together with ADA-positive NAb-negative samples were considered NAb not-positive in specific reports for this paper.

#### 2.3.2 Pharmacokinetic measurements

PK endpoints of ADL included maximum observed serum concentration (C_max_) and area under the serum concentration–time curve over the dosing interval (AUC_tau_) obtained during a 2-week intensive sampling interval (weeks 30−32), time of maximum observed serum concentration (T_max_), average serum concentration (C_av_), apparent clearance (CL/F) as determined from the concentration–time data obtained during the intensive 2-week PK sampling interval. In addition, predose concentrations (C_trough_) were obtained at prespecified PK sampling time points (weeks 10, 16, 22, 24, 26, 28, and 30) (19). ADL serum concentrations between weeks 30 and 32 were analyzed using standard noncompartmental analysis to estimate PK parameters (C_max_, AUC_tau_, T_max_, C_av_, and CL/F) for each individual patient.

PK serum samples were analyzed for ADL at QPS LLC (Newark, Delaware, USA) using a validated analytical ELISA sandwich format assay which measured active ADL including free and active ADL with a lower limit of quantification of 250 ng/mL. In this assay, PF-06410293 or ADL are captured onto a microtiter plate coated with TNFα. The bound PF-06410293 or ADL are detected with the enzyme conjugate, goat anti-human IgG horseradish peroxidase. 3, 3′,5,5′-tetramethylbenzidine is utilized as substrate for signal generation and colorimetric readout.

#### 2.3.3 Safety evaluations

The safety population (safety-randomized) included all randomized patients who received at least 1 dose of investigational product. The safety population was used for the medical evaluation of AEs to identify potential immunologically related AE; these data were further assessed based on ADA and NAb status relative to the AE start date.

During the study’s ongoing safety reviews, all treatment-emergent AEs (TEAEs) were medically evaluated to identify potential unwanted immunogenic AEs, as per FDA (15) and EMA (14) guidelines. AEs were classified using the Medical Dictionary for Regulatory Activities (MedDRA, version 24.0) classification system. All AEs were graded for severity using the NCI Common Terminology Criteria for Adverse Events (CTCAE, version 5.0) (23). A serious AE was pre-defined in the study protocol as any untoward medical occurrence that, at any dose: (a) results in death; (b) is life threatening; (c) requires inpatient hospitalization or prolongation of existing hospitalization; (d) results in persistent disability/incapacity; (e) results in a congenital anomaly/birth defect; (f) other situations.

Medical or scientific judgment was exercised in deciding whether SAE reporting was appropriate in other situations such as important medical events that may not have been immediately life threatening or that did not result in death or hospitalization but may have jeopardized the participant or may have required medical or surgical intervention to prevent one of the other outcomes listed in the above definition. These events were usually considered serious. Examples of such events included invasive or malignant cancers, intensive treatment in an emergency room or at home for allergic bronchospasm, blood dyscrasias or convulsions that did not result in hospitalization, or development of drug dependency or drug abuse.

Anaphylaxis (FDA)/Hyperacute Acute Reactions (EMA): mainly Type I hypersensitivity responses, driven by Th2 phenotypes and characterized by IgE production. Clinical evaluation included fast start of symptoms, 5 to 30 minutes after drug administration, that include skin and mucosal signs and symptoms and respiratory, vascular, and/or gastrointestinal manifestations. These responses may be mild, localized erythema and edema or severe and life-threatening. A careful evaluation of each potential case included assessment of response to treatment.

Cytokine Release Syndrome (CRS) (FDA): a condition where the drug triggers an increased cytokine release that can be from innate or adaptive responses. Using the American Society for Transplantation and Cellular Therapy (ASTCT) classification (24) CRS can be defined by clinical manifestations of fever, hypotension, and hypoxia. Potential cases may be followed testing banked serum samples for cytokine and other mediators, such as C-reactive protein, TNF-α, IL-2, IL-6, IL-10, IFN-γ, and IL-1b, among other cytokines.

Infusion Reactions (FDA)/“non allergic” injection-site and infusion reactions (EMA): these were collected using specific clear instructions for the sites to identify them as injection-site reactions (ISR) and to describe all manifestations of each ISR, such as start and end date, and grade.

Non-Acute reactions (FDA)/Delayed reactions (EMA): classified as Hypersensitivity Type III and are characterized by Immune Complex-mediated responses involving IgM or IgG. As ADL has been shown to form high molecular weight complexes with the target, which could lead to large complex formation with ADA (25). Type IV hypersensitivity reactions are T cell driven and are not antibody dependent. This is also known as delayed type hypersensitivity and is characterized by local or systemic signs and symptoms secondary to innate and adaptive cytokine secretion, often with complement activation. Medical evaluation of all AEs was conducted to identify the most common signs and symptoms of potential immunologically related delayed/non-acute immune response, with a delay in symptoms (1−2 weeks following the administration). In addition, banked serum was available for complement levels if needed.

AEs programmatically identified using narrow and broad Standardized MedDRA Queries (SMQ) for hypersensitivity, angioedema, and anaphylactic reactions underwent regular medical evaluation to identify potential immunologically related AEs. In addition, AEs that fulfilled the Sampson Criteria underwent medical evaluation to identify potential anaphylactic reactions to the drug or triggered by the drug (26). The following data were considered: date and time of dose before AE start; patient clinical features before, during, and after the AE, such as response to repeat doses, grade of AE, outcome, and response to treatment for the AE; and signs and symptoms reported for each potential immunologically related AE were considered, as recommended in the EMA and FDA guidelines (14, 15). All safety data were summarized descriptively.

AEs including ISRs were managed by the investigators following their local standard of practice.

#### 2.3.4 Statistical analysis

PK endpoints were summarized descriptively and by ADA and NAb status at week 30. The PK population included all randomized patients who received treatment to initiate the week 30 steady-state PK profile, remained on background MTX with no major protocol deviations influencing the PK assessment, and had achieved AUC_tau_ or C_max_. The PK population was used for week 30 to 32 PK analysis (AUC_tau_, C_max,_ T_max_, C_av_, C_trough,_ and CL/F).

For the ADL ADA ECL immunoassay, statistical analyses were completed using JMP Statistical Discovery Software (Version 10.0; SAS Institute, Inc., Cary, NC, USA).

For the ADA and NAb data, the percentage of patients with positive ADA and positive NAb was calculated and summarized descriptively in the safety-randomized population by each visit. The magnitude (titer) and the time of the first positive sample were also described. The time of first positive ADA sample after baseline (day 1) was assessed by the presence or absence of ADA at baseline, as pre-existing ADA is considered a potential risk of ADA titer booster after dosing (27). If the last available sample for a patient was positive for ADA or NAb, the patient’s ADA and NAb response was characterized as persistent.

The impact of ADA and NAb on PK over time was also assessed and summarized descriptively. The median C_trough_ PK concentrations at each study visit during the study duration for the safety-randomized population were summarized by the corresponding ADA status (positive and negative) at each visit. The PK concentrations from weeks 30 to 32 were presented by ADA status at week 30 (last dose before PK sampling during those weeks).

## 3 Results

### 3.1 ADA and NAb

All drug concentrations measured were within the drug tolerance limits of the ADA assay. Of the 427 patients, 250 (58.5%) had at least 1 ADA-positive sample, and 221 (221/427; 52%) had persistent ADA. Of the 250 patients with ADA, 61 had NAb (61/427 total patients; 14.3%, and 42 of these 61 patients (42/427 total patients; 9.8%) had persistent NAb (**Table 1**).

**Table 1:** Patients with persistent ADA or NAb response up to week 32 (safety-randomized population).

	Total N = 427
ADA-positive, n	250 (58.5%)
ADA persistent/ADA-positive, n/N (%)	221/250 (88.4%)
ADA persistent/total, n/N (%)	221/427 (51.8%)
NAb-positive, n	61 (14.2%)
NAb persistent/NAb-positive, n/N (%)	42/61 (68.9%)
NAb persistent/total, n/N (%)	42/427 (9.8%)

ADA, antidrug antibody; N, number of patients in safety analysis set; n, number of patients with ADA-positive/NAb-positive samples/ADA/NAb persistent response (ADA-positive at last available sample/NAb-positive at last ADA-positive sample); NAb, neutralizing antibody.

ADA-/NAb-positive responses were counted at any visit including baseline (day 1). ADA-positive: ADA titer ≥1.88; ADA-negative: ADA titer <1.88. NAb-positive: NAb titer ≥0.7; NAb-negative: <0.7.

Among the 417 patients who were ADA-negative at baseline, 43.1% (180/417) developed ADA against ADL by week 16 (**Table 2**). All 10 patients who had ADA-positive samples at baseline had a first positive ADA sample detected after randomization (incidence by visit) within 16 weeks of starting treatment (**Table 2**). Of the 10 patients with ADA-positive samples at baseline, 1 was also NAb-positive and 9 were NAb-negative (data not shown). Regardless of pre-existing ADA, 61 of 250 patients who had ADA had NAb; the first NAb-positive sample was detected within the first 16 weeks of treatment in most of the 61 (~80%) patients who had NAb-positive ADAs (**Table 2**).

**Table 2:** Time of the first positive ADA and positive NAb sample at randomization (week 10) and beyond by visit (safety-randomized population for patients with ADA-positive / ADA-negative samples at baseline).

Time of first ADA-positive sample by week	ADA-positive at baseline (N=10)	ADA-negative at baseline (N=417)
Week 10, n (%)	8 (80.0)	106 (25.4)
Week 16, n (%)	2 (20.0)	74 (17.7)
Week 22, n (%)	0	27 (6.5)
Week 24, n (%)	0	7 (1.7)
Week 26, n (%)	0	7 (1.7)
Week 28, n (%)	0	9 (2.2)
Week 30, n (%)	0	4 (1.0)
Week 32, n (%)	0	6 (1.4)
Time of first NAb-positive sample by week	ADA-positive at baseline (N=10)	ADA-negative at baseline (N=417)
Week 10, n (%)	4 (40)	37 (8.9)
Week 16, n (%)	0	8 (1.9)
Week 22, n (%)	0	4 (1.0)
Week 24, n (%)	0	2 (0.5)
Week 26, n (%)	0	1 (0.2)
Week 28, n (%)	0	0
Week 30, n (%)	0	2 (0.5)
Week 32, n (%)	0	3 (0.7)

ADA, antidrug antibody; n, the number of patients who were first identified as having an ADA-positive or NAb-positive sample at the corresponding visit; N, the number of patients who had an ADA-positive/-negative sample at baseline (day 1). Early termination visit was not included. ADA-/NAb-positive responses were counted at any visit including baseline (day 1). ADA-positive: ADA titer ≥1.88; ADA-negative: ADA titer <1.88. Positive ADA samples were tested for neutralizing activity (NAb). NAb-positive: titer ≥0.7; NAb-negative: titer <0.7.

**Figure 1:**
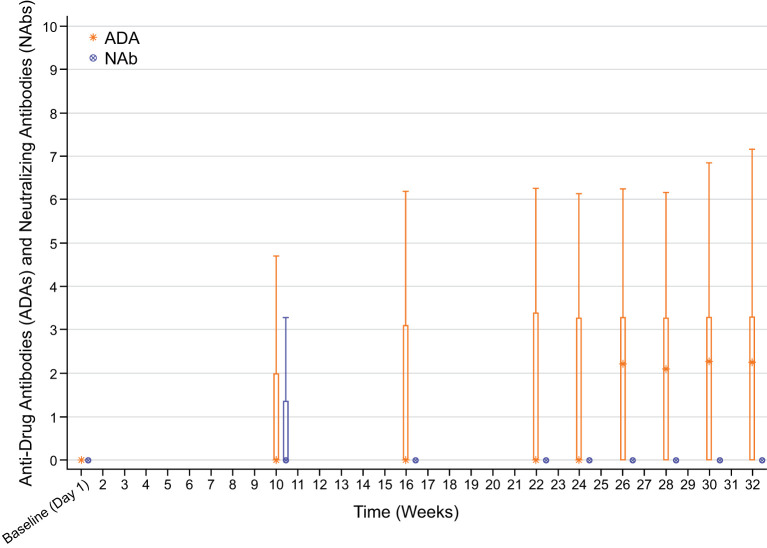
ADA and NAb titers by visit (safety-randomized population). Summary statistics have been calculated by setting values below the lower limit of quantification (<1.88) to zero. Early Termination visit is not included. Box plot provides median values (represented by stars and dots) and 25%/75% quartiles with whiskers to the last point within 1.5 times the interquartile range.

**Figure 1** shows that median ADA titers by visit were stable by week 16 with no titer boosters. NAb titers remained stable from week 16 until week 32.

### 3.2 Pharmacokinetics

**Figure 2:**
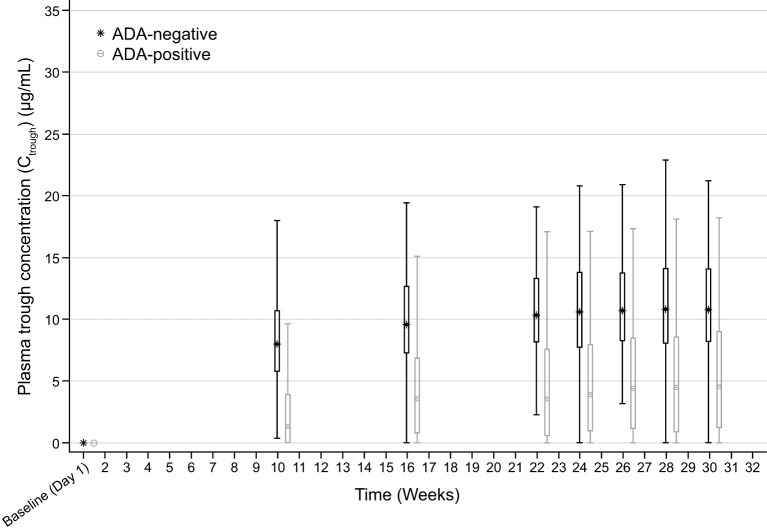
Box plot of C_trough_ by visit and ADA status (safety-randomized population). ADA, antidrug antibody. Early Termination visit is not included. Box plot provides median values (represented by stars and dots) and 25%/75% quartiles with whiskers to the last point within 1.5 times the interquartile range. ADA-positive: ADA titer ≥1.88; ADA-negative: ADA titer <1.88. C_trough_ at each visit is summarized by ADA status at the corresponding visit. Summary statistics have been calculated by setting concentration values below the lower limit of quantification to zero. The lower limit of quantification is 0.25 ug/mL.

**Figure 3:**
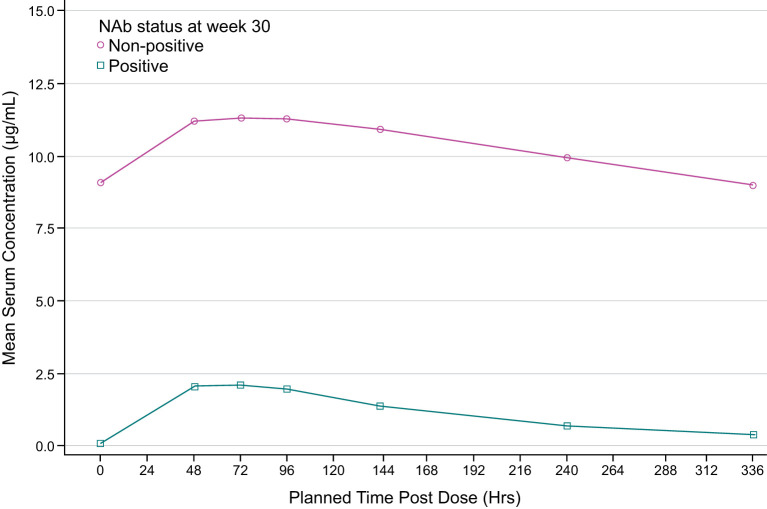
Mean pharmacokinetic (PK) concentrations time plot (linear scale) by NAb status at week 30 (PK population). ADA, antidrug antibody; NAb, neutralizing antibody. NAb-positive: NAb titer ≥0.7. The lower limit of quantification is 0.25 ug/ml. Summary statistics have been calculated by setting concentration values below the lower limit of quantification to zero. NAb non-positive group includes the patients with ADA-positive/NAb-negative and ADA-negative not tested for NAb activity.

Descriptive analyses of PK endpoints demonstrated lower median ADL C_trough_ concentrations during the study in ADA-positive samples as compared with ADA-negative samples (**Figure 2**). Furthermore, lower median ADL AUC_tau_, C_max_, C_av_, and higher CL/F levels were observed between weeks 30 and 32 in patients with ADA-positive as compared with ADA-negative samples at week 30 (**Table 3**). The potential impact of the ADA-positive samples with neutralizing activity (NAb-positive) on ADL PK concentrations appeared to be more pronounced as compared with NAb-non-positive samples (ADA-positive/NAb-negative and ADA-negative not tested for NAb activity) (**Figure 3**).

**Table 3:** AUC_tau_, C_max_, T_max_, C_av_, and CL/F in ADA-positive and ADA-negative patients at week 30 (PK population).

Parameter (Units)	ADA-negative (N=186)						
	n	Geometric Mean	Geometric CV (%)	Arithmetic Mean	SD	CV (%)	Median (range)
AUC_tau_ (µg*hr/mL)	183	3788	52	4178	1669.8	40	4030 (340−10700)
C_max_ (µg/mL)	186	13.27	45	14.45	5.7814	40	13.85 (3.27−37.3)
T_max_ (hr)	186	–	–	–		–	71.60 (0−335)
C_av_ (µg/mL)	183	11.27	52	12.44	4.9686	40	12.00 (1.01−31.8)
CL/F (mL/hr)	183	10.56	52	12.44	11.548	93	9.930 (3.75−118)
Parameter (Units)	ADA-positive (N=194)						
	n	Geometric Mean	Geometric CV (%)	Arithmetic Mean	SD	CV (%)	Median (range)
AUC_tau_ (µg*hr/mL)	189	1567	169	2370	1657.7	70	2280 (11.5−6930)
C_max_ (µg/mL)	194	6.290	115	8.508	5.4778	64	7.885 (0.267−24.1)
T_max_ (hr)	194	–	–	–	–	–	72.30 (0−337)
C_av_ (µg/mL)	189	4.663	169	7.053	4.9329	70	6.790 (0.0343−20.6)
CL/F (mL/hr)	189	25.53	169	91.46	352.87	386	17.50 (5.77−3470)

ADA, antidrug antibody; AUC_tau_, area under serum concentration–time curve over dosing interval; C_av_, average serum concentration; CL/F, apparent clearance; C_max_, maximum observed serum concentration; CV, coefficient of variation; T_max_, time of maximum observed serum concentration; N, total number of patients in the treatment group; n, number of patients contributing to summary statistics; PK, pharmacokinetics. ADA-positive: ADA titer ≥1.88; ADA-negative: ADA titer <1.88. C_trough_ at each visit is summarized by ADA status at the corresponding visit.

### 3.3 Safety

Immunologically related AEs were mostly ISRs and some hypersensitivity reactions, which are summarized for patients by their ADA-positive or ADA-negative status at week 10 (**Table 4**). Of the 114 patients with an ADA-positive sample at week 10, 4 patients were identified by medical evaluation with potential immunologically related AEs (**Table 4**). All 4 patients had a corresponding positive ADA sample at the time of their event. One of these patients had 7 repeat ISRs and 1 hypersensitivity event of perioral dermatitis reported as mild (Grade 1), which responded to topical zinc ointment. Two patients reported 1 ISR each with a corresponding ADA-positive sample. The fourth patient of the 114 total patients with positive ADA at week 10 had a hypersensitivity qualifying event of pruritic rash (Grade 1) that responded to antihistamines and had a corresponding ADA-positive sample at week 30. This patient permanently discontinued the study.

**Table 4:** Summary of patients with medically evaluated potential immunologically related AEs during week 10 and beyond (Safety population ADA-positive / ADA-negative by week 10).

ADA-positive at week 10 (N=114)						
Patient	Visit	Study day of ADA / NAb sample assessment	ADA titer	NAb titer	Potential immunogenic event	Study day of AE start
1	Week 30	211	4.18	2.39	Hypersensitivity	214
2	Week 24	169	4.22	2.12	ISR	183
3	Week 10	71	5.11	3.19	ISR	71
4	Week 16	113	3.67	<0.70	ISR	115
	Week 22	155	3.74	<0.70	ISR	158
	Week 24	170	4.12	<0.70	ISR	172
	Week 24	170	4.12	<0.70	Hypersensitivity	182
	Week 26	184	3.78	<0.70	ISR	187
	Week 28	197	4.07	<0.70	ISR	199
	Week 30	211	3.99	<0.70	ISR	213
	Week 32	225	4.18	<0.70	ISR	227
ADA-negative at week 10 (N=311)						
Patient	Visit	Study day of ADA / NAb sample assessment	ADA titer	NAb titer	Potential immunogenic event	Study day of AE start
1	Week 10	69	<1.88		ISR	85
	Week 16	111	<1.88		ISR	112
	Week 16	111	<1.88		ISR	126
	Week 16	111	<1.88		ISR	139
2	Week 16	113	<1.88		ISR	114
3	Week 16	113	2.77	<0.70	ISR	141
4	Week 22	155	2.76	<0.70	ISR	156
5	Week 10	71	<1.88		Hypersensitivity	102
6	Week 16	114	<1.88		ISR	141
7	Week 10	70	<1.88		ISR	72
	Week 10	70	<1.88		ISR	84
	Week 10	70	<1.88		ISR	142
	Week 16	114	<1.88		ISR	128
	Week 16	114	<1.88		ISR	141
	Week 16	114	<1.88		ISR	155
	Week 22	155	<1.88		ISR	169
	Week 26	183	<1.88		ISR	184
	Week 26	183	<1.88		ISR	197
	Week 28	197	2.37	<0.70	ISR	211
	Week 30	211	2.55	<0.70	ISR	225
8	Week 10	71	<1.88		ISR	101
9	Week 10	75	<1.88		ISR	86
	Week 10	75	<1.88		ISR	100
	Week 16	113	<1.88		ISR	127
	Week 22	155	<1.88		ISR	169
	Week 24	169	<1.88		ISR	183

ADA, antidrug antibody; AE, adverse event; ISR, injection-site reaction; NAb, neutralizing antibody.

ADA-positive: ADA titer ≥1.88; ADA-negative: ADA titer <1.88.

NAb-positive: NAb titer ≥0.7; NAb-negative: <0.7.

Visit was the corresponding visit of the ADA/NAb sample date. For immunologically related AE, the corresponding ADA status (positive or negative) was the most recent available ADA result before the AE start date (not the same visit day).

For ISR, the corresponding ADA status (positive or negative) was the ADA result immediately before the ISR (on same visit day); if not available, the previously available ADA result was used.

Of the 311 patients with an ADA-negative sample at week 10, 8 patients reported ISRs and 1 additional patient had a medically evaluated immunologically related hypersensitivity reaction. Repeat ISRs were reported in 3 of the 8 patients who had ISRs. Two of these patients who reported repeat ISRs had corresponding ADA-negative samples. The third patient with repeat ISRs had corresponding ADA-negative results at the time of the ISRs, except for those ISRs on week 30 and week 32 who had corresponding ADA-positive results at weeks 28 and 30 (**Table 4**). Three of the 5 patients who reported non-repeat ISRs had corresponding ADA-negative samples, and 2 patients had had a corresponding ADA-positive sample (**Table 4**). Only 13/427 (3%) patients had an immunologically related AE irrespective of the presence of ADA. One patient had dermatitis Grade 1, which started 3 days after a dose, that required steroids and did not recur with future injections. There were no cases of anaphylaxis, angioedema, cytokine release syndrome, or delayed immune responses. None of the ISR and hypersensitivity reactions were severe as per the CTCAE grading system, or serious as defined in the protocol, and all resolved, most with no treatment needed.

Anaphylaxis/Hyperacute Acute Reactions: There were 3 cases of potential immunologically related hypersensitivity reactions, all Grade 1 (mild), and presented only once for each patient, which did not recur with repeat doses. Two had pre-existing and persistent ADA, and the third case had no ADA. These manifestations resolved with steroid or antihistamine treatment, and 1 case with zinc ointment for perioral dermatitis. No case fulfilled the Sampson criteria for anaphylaxis (26).

Cytokine Release Syndrome (CRS) (24): There were no potential CRS AEs identified in this study. No case presented with elevated body temperature; a condition required for CRS ASTCT consensus grading.

Infusion Reactions/“non allergic” injection-site and infusion reactions (ISR): ISR were reported by 11 patients, either just once or multiple times after drug administration. The preferred terms reported for each ISR included erythema, induration, injection site pain, edema, pruritus and swelling. All were Grade 1 (mild) and resolved within a day.

Non-Acute reactions/Delayed reactions: No cases of delayed immune response were identified in this study.

## 4 Discussion

In this study, we performed systematic evaluations to characterize the immunogenicity of ADL and assessed the potential relationship between presence of ADA and PK (as a surrogate of efficacy) and immunologically related AEs in patients who participated in a randomized, controlled, phase 3 interchangeability study. To our knowledge, this is the first time an analysis of this type has been conducted. With the increasing availability of biologic therapies, it is important to evaluate unwanted immune responses and their potential impact on PK, efficacy, and safety (14, 20). This information is critical for regulatory agencies to make decisions on the development of the therapeutic as well as to implement strategies to mitigate potential immunogenic responses with clinical significance (14, 20). To do this, regulatory agencies and practicing physicians need a comprehensive and thorough assessment of the immunogenicity observed during the development of biologic therapeutics.

Assessing potential associations between immunologically related AEs and ADA status requires an appropriate strategy for timing of sampling for ADA measurement, appropriate collection, and assessment of AEs with frequent medical evaluation for identification of potential immunologically related AEs, and the use of validated, sensitive, and drug and target-tolerant ADA assays. In particular, ADA assays need to show drug tolerance higher than observed drug concentrations at the time of ADA sampling (typically C_trough_) to be able to reliably detect ADA in samples containing high levels of drug and avoid false-negative results. Assays have become increasingly reliable in this regard over the last decade; however, care should be taken to ensure ADA assay performance characteristics are understood and suitable for their application. In this study, we used an assay demonstrating drug tolerance of up to 150 μg/mL, whereas the highest observed C_max_ concentrations during study were <38 μg/mL. Thus, the likelihood of false-negative ADA results is assumed to be low.

In our cohort, over half (59%) of patients receiving ADL developed ADA against ADL, most within the first 3 months of dosing. Over half (52%) of patients had persistent ADA and <10% had persistent NAb. These percentages of patients with ADA-positive samples are consistent with previous reports (17, 28, 29). The molecular mechanisms that lead to the development of ADA are thought to negatively correlate with the “degree of humanness” of a protein therapeutic (30). As ADL is a humanized antibody, low clinical immunogenicity was originally expected for this drug. However, it should be noted that the active binding site/complementarity-determining region (CDR) of ADL is not fully human. Consistent with this, ADL exhibits immunogenicity in almost, if not all, reported clinical studies. It is now generally accepted that most ADA against therapeutic antibodies will develop against the CDR of the drug (i.e., anti-idiotypic antibodies), which is unlikely to be present in the B cell repertoire of most individuals.

In this study, PK was used as a surrogate of efficacy and further exploration of PK and its relationship to ADA-positivity and NAb-positivity was evaluated. ADA can alter the PK of a biological treatment by affecting clearance, and thus either shortening or lengthening the elimination half-life of the biological treatment (31). In addition, in some cases ADA can decrease treatment efficacy by binding to the drug molecule in a manner which neutralizes the activity of the drug (neutralizing antibody, NAb), while other ADA may be non-neutralizing. In the current study, ADA was measured using a validated, highly sensitive and drug-tolerant ECL assay and ADA-positive samples were tested for neutralizing and non-neutralizing antidrug antibodies using a validated cell-based assay. Results demonstrated that ADA appeared to increase ADL mean CL by approximately 2-fold in patients with ADA-positive samples as compared to patients with ADA-negative samples at week 30, leading to an approximate 2-fold decrease in exposures (AUC_tau_ and C_max_). These results are consistent with previous reports of the impact of ADA development on PK in anti-TNFα treated patients (32). Notably, the impact of ADA on ADL exposures was much more pronounced in patients with ADA-positive samples with NAb (ADA-positive/NAb-positive) than in patients with NAb-non-positive samples (ADA-positive/NAb-negative and ADA-negative not tested for NAb activity). ADA-positive/NAb-positive AUC_tau_ and C_max_ were approximately 15-fold and 7-fold lower, respectively as compared to patients with NAb-non-positive samples.

ISR was the most frequently reported immunologically related AE (n = 11), although all were mild (Grade 1) and resolved within a day. The mechanisms implicated in the development of hypersensitivity reactions with ADL involve Type I, III, and IV reactions, with ISR being caused by a Type I reaction and the most common adverse reaction to subcutaneous biologicals (6). ADL has been characterized as an immunogenic drug, which we confirmed in this study with more than half the patients developing ADA to the drug. However, the presence of ADA was not associated with immunogenically-related AEs including hypersensitivity reactions (acute or delayed) and ISR. The development of acute hypersensitivity and ISRs in different individuals was observed to be independent of their ADA status at start of AE. Of the patients with multiple ISRs, 1 was ADA-positive, and 3 were ADA-negative. There were 3 cases of potential immunologically related hypersensitivity reactions that were all mild (Grade 1), and presented only once for each patient, and did not recur with repeat doses. Two had pre-existing and persistent ADA, and the third case had no ADA. No case fulfilled the Sampson criteria for anaphylaxis (26).

Hence, while more than half of the ADL-treated patients developed ADA, we found no clinical impact of ADA on safety (ISRs and hypersensitivity). Moreover, pre-existing ADA to ADL by week 10 (lead-in period with ADL-REF) did not predispose patients to immunologically related AEs after randomization. This, to our knowledge, is the first comprehensive study that evaluated the safety with attention to the design on ADA sample collection versus drug administration and safety data collection. For the prescriber and the patient, the conclusions from this study support that the clinical identification and management of all potential immunologically related AEs do not require ADA testing, and that the AE is more likely to be manifested as a mild ISR. Treatment of these events should be as per local guidelines. Our study confirmed that the likelihood of CRS and delayed immune responses due to immune complex deposition or T cell activation is low or none for ADL which remains with a favorable risk/benefit profile for patients with immune diseases.

In summary, this analysis included a robust dataset of patients with multiple PK and safety assessments obtained at known time points to relate to ADA measurements. Over 50% of patients developed ADAs to ADL during the study. The presence of ADA with NAb occurred in <10% of patients who received the drug and had an impact on ADL PK characteristics, resulting in lower drug concentrations and increased clearance. Of the patients who developed an ADA response or had pre-existing ADA against ADL, there was no association with the development of immunologically related AEs.

## Data Availability

The original contributions presented in the study are included in the article/supplementary materials. Further inquiries can be directed to the corresponding author. Upon request, and subject to review, Pfizer will provide the data that support the findings of this study. Subject to certain criteria, conditions, and exceptions, Pfizer may also provide access to the related individual de-identified patient data. See https://www.pfizer.com/science/clinical-trials/trial-data-and-results for more information.
